# Supplementary material for: Biodegradable polyphosphoester micelles act as both background-free 31P magnetic resonance imaging agents and drug nanocarriers
Source: Nat Commun. 2023 Jul 19;14:4351. doi: 10.1038/s41467-023-40089-0 (PMC10356825; doi:10.1038/s41467-023-40089-0)
Supplement: Supplementary file 1 — Supplementary Information [file 41467_2023_40089_MOESM1_ESM.pdf]

Supplementary Information

Biodegradable Polyphosphoester Micelles Act as Both Background-free ^{31}P Magnetic Resonance Imaging Agents and Drug Nanocarriers

Olga Koshkina[1]*, Timo Rheinberger[1], Vera Flocke[2], Anton Windfelder[3,4], Pascal Bouvain[2], Naomi M. Hamelmann[5], Jos M.J. Paulusse[5], Hubert Gojzewski[1], Ulrich Flögel[2]*, Frederik R. Wurm[1]*

Affiliations:

[1]Sustainable Polymer Chemistry Group, Department of Molecules and Materials, MESA+ Institute of Nanotechnology, University of Twente, Enschede, The Netherlands.

[2]Department of Molecular Cardiology, Experimental Cardiovascular Imaging, Heinrich-Heine-University, Düsseldorf, Germany.

[3]Department of Bioresources, Fraunhofer Institute for Molecular Biology and Applied Ecology IME, Giessen, Germany

[4]Laboratory of Experimental Radiology, Justus Liebig University, Giessen

[5]Biomolecular Nanotechnology Group, Department of Molecules and Materials, MESA+ Institute of Nanotechnology, University of Twente, Enschede, The Netherlands.

*Corresponding authors. Email: o.koshkina@utwente.nl, floegel@uni-duesseldorf.de, frederik.wurm@utwente.nl

OK & TR contributed equally

1 Synthesis and characterization of polyphosphoesters and colloidal nanostructures for ^{31}P MRI **3**
1.1 Nanoparticles from a hydrophobic PPn 3
1.2 Overview of all polymers 3
1.3 Copolymerization kinetics of gradient copolymer with ethyl- and phenyl- side chains (Ph-*grad*-Et-PPn polymer) 4
1.4 Size exclusion chromatography (SEC) of Ph-*grad*-Et-PPn copolymers 7
1.5 Determination of T_g of PPn gradient copolymers 7
1.6 NMR spectroscopy of PPn_{GRAD} micelles 8
1.7 Critical Micelle Concentration (CMC) 8
1.8 Visualization of PPn_{GRAD} micelles by Atomic Force Microscopy (AFM) 9
1.9 Stability of PPn_{GRAD} micelles 10
1.10 Viability assay with immune cells using PPn_{GRAD} micelles 10
2 ^{31}P MRI: characterization of MR properties, *in vitro* and *in vivo* imaging **12**
2.1 Discussion of relaxation times measured by ^{31}P NMR spectroscopy 12
2.2 Determination of ^{31}P MRI signal-to-noise ratio 14
2.3 ^{31}P MRI in vivo 15
2.4 Degradation of PPn_{GRAD} micelles in vivo 16
3 Experimental details **16**
3.1 Materials 16
3.2 Synthesis 17
3.2.1 Monomer syntheses 17
3.2.2 Polymerizations 19
3.2.3 Preparation of Colloidal Dispersions for Imaging 20
3.3 Magnetic Resonance Imaging 21
3.4 Cell Experiments 22

1 Synthesis and characterization of polyphosphoesters and colloidal nanostructures for ^{31}P MRI

1.1 Nanoparticles from a hydrophobic PPn

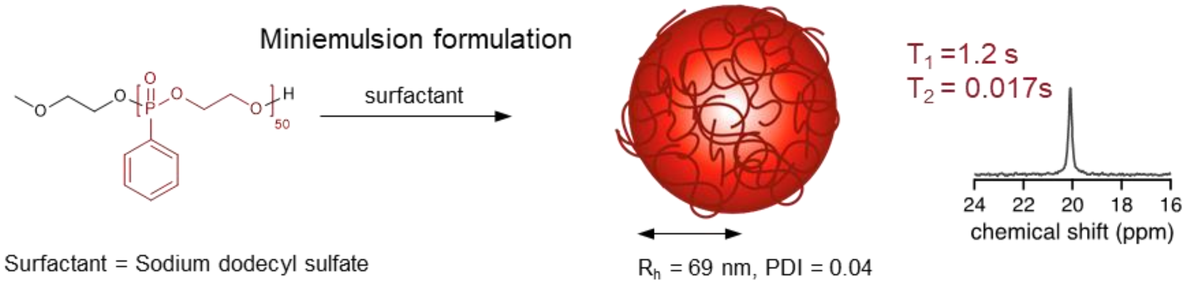

Supplementary Fig. 1. Nanoparticles from hydrophobic PPn with phenyl side group. Ph-PPn was formulated to nanoparticles using miniemulsion technique with sodium dodecyl sulfate (SDS) as surfactant, resulting in nanoparticles of 69 nm radius and a low polydispersity index (PDI). The nanoparticles displayed an NMR signal (H_2O/D_2O 9:1 v:v, 158 MHz). The longitudinal relaxation time T_1 was suitable for imaging; however, the transverse relaxation time T_2 was too short for conventional imaging sequences, such as spin-echo sequence.

1.2 Overview of all polymers

Supplementary Table 1. Summary of polymer properties; resulting colloidal dispersion indicated in the brackets.

Sample	M_n [a] (g mol^{-1})	M_w [a] (g mol^{-1})	Đ [a]	$P_{n,theo}$ (Ph-Pn)	$P_{n,exp}$ [b] (Ph-Pn)	$P_{n,theo}$ (Et-Pn)	$P_{n,exp}$ [b] (Et-Pn)
**Ph-PPn**	9200	10700	1.16	50	50	-	-
**Ph-PPn-b-PEG** (PPn_{CORE})	10400	14100	1.35	30	30	-	-
**PS-b-Et-PPn** (PPn_{SHELL})	35500	44800	1.25	-	-	62	62
**Ph-grad-Et-PPn** batch 1 (PPn_{GRAD})	7900	8500	1.08	30	30	30	30
**Ph-grad-Et-PPn** batch 2 (PPn_{GRAD})	6000	6800	1.14	30	32	30	28
**Ph-grad-Et-PPn** batch 3 (PPn_{GRAD})	6400	7200	1.12	30	32	30	33

[a]Determined by SEC, [b]determined by NMR spectroscopy (backbone integral against initiator integral, $CDCl_3$, 400 MHz). Ph-grad-Et-PPn batch 1 was used on Fig. 2F, G, batch 2 on Fig 3 C,D, and Fig. 4, and batch 3 for Fig. 3E and

1.3 Copolymerization kinetics of gradient copolymer with ethyl- and phenyl-side chains (Ph-grad-Et-PPn polymer)

We performed statistical copolymerizations of 2-ethyl-2-oxo-1,3,2-dioxaphospholane (Et-Pn) and 2-phenyl-2-oxo-1,3,2-dioxaphospholane (Ph-Pn) in dichloromethane (DCM) with a metal-free superbase 1,8-diazabicyclo[5.4.0]undec-7-ene (DBU) as the respective catalyst (Fig. 2A, main text). To follow the kinetics, the copolymerization was, additionally, conducted directly in an NMR tube. The comonomer consumption was followed by real-time ^{31}P NMR spectroscopy. From the real-time NMR measurements, we calculated the reactivity ratios for Et-Pn and Ph-Pn using four different nonterminal models, as proposed by Jaacks,[1] BSL,[2] and Frey[3] (ideal integrated model) and the Meyer-Lowry[4] (Supplementary Fig. 1). All four models resulted in similar reactivity ratios under these conditions, r(Ph-Pn)= 23.9 ± 0.6 and r(Et-Pn)= 0.040 ± 0.002, for fitting up to 40 % of the total monomer conversion (Supplementary Table 2). The determined reactivity ratios from the ideal integrated model were used to calculated the polymer fraction vs. conversion (main text, Fig. 2 B); a Monte Carlo simulation was performed as reported previously (main text, Fig. 2 C,D).[5] This visualization of the copolymerization, clearly shows the formation of a gradient structure in the final macromolecule.

Monte Carlo simulations

Monte Carlo simulations were performed in MATLAB R2020b.

First the reactivity ratios r_A and r_B were set to the calculated values. $f_{A,0}$ was set to the initial fraction of monomer A. Each simulation was performed with 10^6 chains (n_{chains}), the PDI was set to one (we set the molar mass dispersity to unity to exclude chain length effects on the visualization). The total number of monomer molecules is set to the number of chains multiplied with the degree of polymerization (DP). The final degree of polymerization was DP=100.

$n_{chains}=10^6$

$n_A= n_{chains}\cdot DP\cdot f_{A,0}$

$n_B= n_{chains}\cdot DP\cdot (1-f_{A,0})$

A chain k is selected by sequence. If the chain k has DP = 0, then: A random number w in the interval [0,1] is created and the probability of incorporation of A is calculated with the Mayo-Lewis equation:

$$F_A = \frac{r_A f_A^2 + f(1 - f_A)}{r_A f_A^2 + 2f_A(1 - f_A) + r_B(1 - f_A)^2}$$

If F>w then add monomer A to the chain, otherwise monomer B.

If chain k has a DP>0, then:

A random number w in the interval [0,1] is created. The monomer at the chain end k is called e. The reactivity ratio corresponding to this chain k was used to calculate the probability of addition of the same monomer as e: $F_e = \frac{r_e f_e}{r_e f_e - f_e + 1}$

If F_e>w then add monomer equal to e, otherwise use the monomer unequal to the chain end.

Then the number of consumed monomers n_x is reduced by 1. Then go to the step, where the next chain k is selected by sequence (➔ PDI=1). These steps are repeated until DP=100.

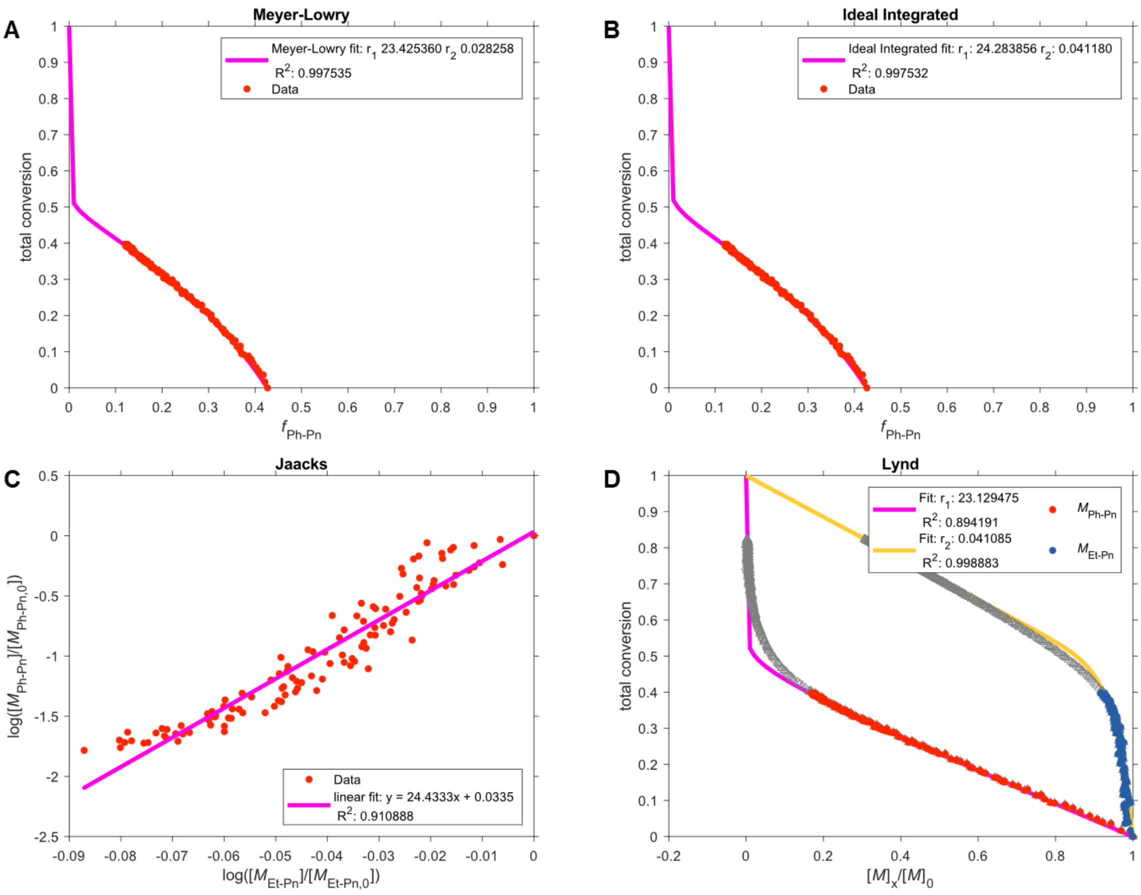

Supplementary Fig. 2. Copolymerization of monomers Et-Pn and Ph-Pn in DCM using 2-(methoxy)ethanol as initiator and DBU as catalyst at -10 °C; for the fitting data up to 40 % of total conversion were used for all three models. Fitting of the real-time NMR data of monomer consumption vs monomer is shown: (A) Mayer-Lowry model, (B) Frey model, (C) Jaacks model, (D) BSL fit on real-time NMR data of monomers. The deviation between reactivity ratios determined by the different models was bellow 5%.

Supplementary Table 2. Reactivity ratios determined from four different nonterminal models and the averaged values

Method	r(Et-Pn)	r(Ph-Pn)
Meyer-Lowry	0.028	23.4
Frey	0.041	24.3
Jaacks	0.037	24.4
BSL	0.041	23.1
Average	0.040 ± 0.002	23.9 ± 0.6

1.4 Size exclusion chromatography (SEC) of Ph-grad-Et-PPn copolymers

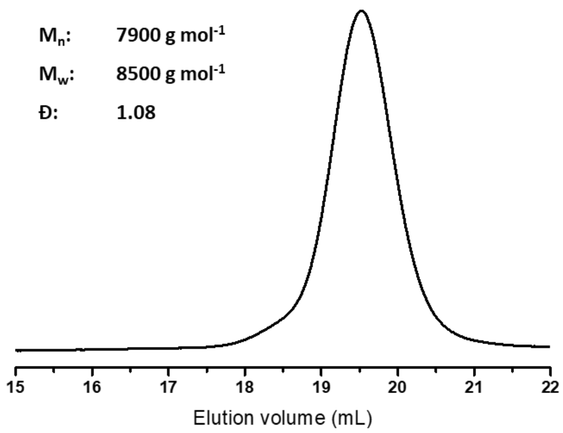

Supplementary Fig. 3. Exemplary SEC elugram of Ph-grad-Et-PPn copolymer showing a narrow distribution (DMF, 50 °C).

1.5 Determination of T_g of PPn gradient copolymers

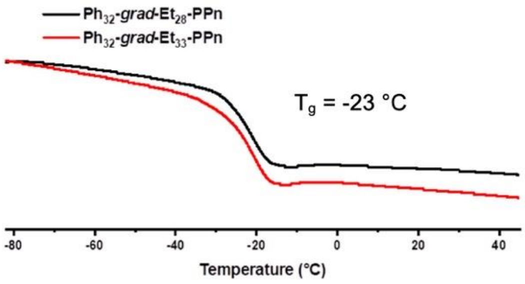

Supplementary Fig. 4. Differential Scanning Calorimetry (DSC) measurements of Ph-grad-Et-PPn copolymer showing the second heating curve (rate of 10 °C min-[1]).

1.6 NMR spectroscopy of PPn_{GRAD} micelles

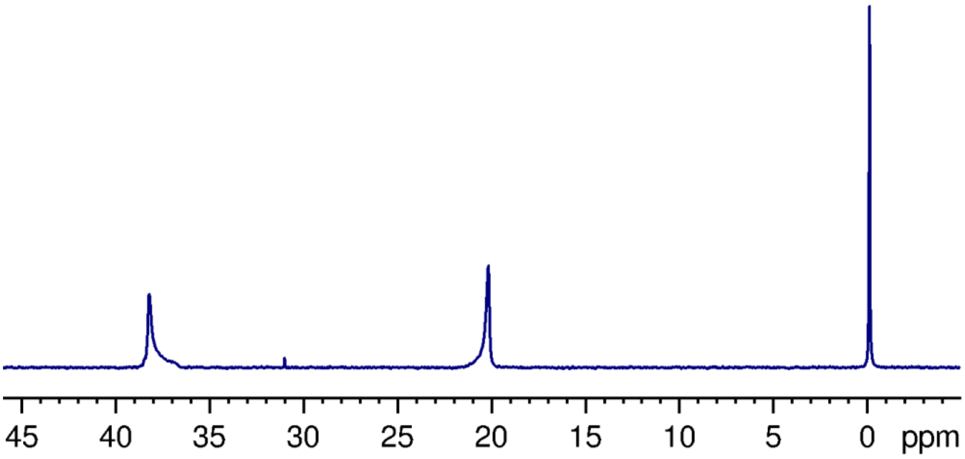

Supplementary Fig. 5. ^{31}P NMR spectrum of PPnGRAD (produced with Ph-grad-Et-PPn batch 1) with sodium phenylphosphate (0 ppm) as a reference. The signal of the reference is symmetric; conversely, the signals of gradient copolymer display an asymmetric peaks shape. In gradient copolymers, a monomer units can have different neighboring monomers units, resulting in different electronic environment and eventually leading to peak asymmetry (H_2O/D_2O 9:1 v:v, 158 MHz).

1.7 Critical Micelle Concentration (CMC)

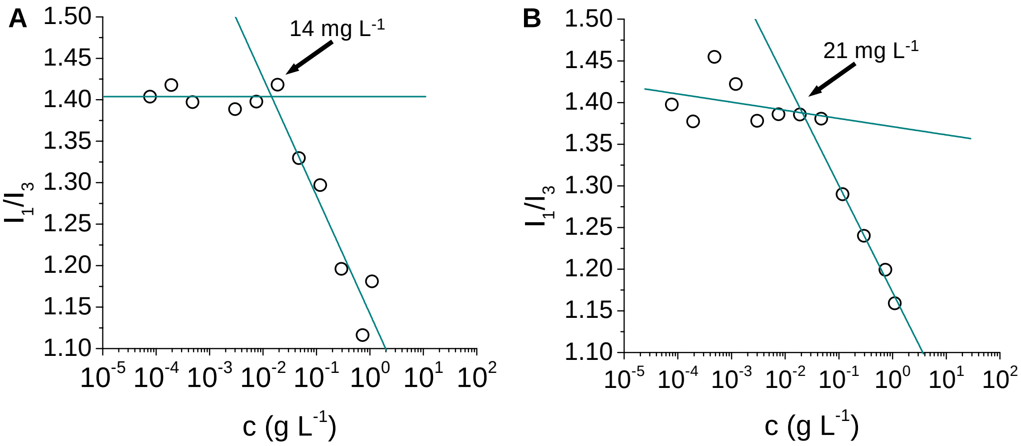

Supplementary Fig. 6. Determination of CMC of gradient copolymer Ph-grad-Et-PPn and block copolymer PPn-b-PEG by pyrene assay. The intensity ratios I_1 (372.4 nm) and I_3 (384.2 nm) are plotted vs concentration c. (A) Ph_{32}-grad-Et_{28}-PPn (batch 2) and (B) Ph-PPn_{30}-PEG.

The CMC was measured using pyrene assay, as described in literature (Fig. S3).[6] The low CMC of the micelles suggests that the micelles should remain stable upon intravenous administration and subsequent dilution in the blood flow. The CMC values are in a comparable

range as CMC of the other copolymers, for example different Pluronic, which are extensively used for drug delivery.

1.8 Visualization of PPnGRAD micelles by Atomic Force Microscopy (AFM)

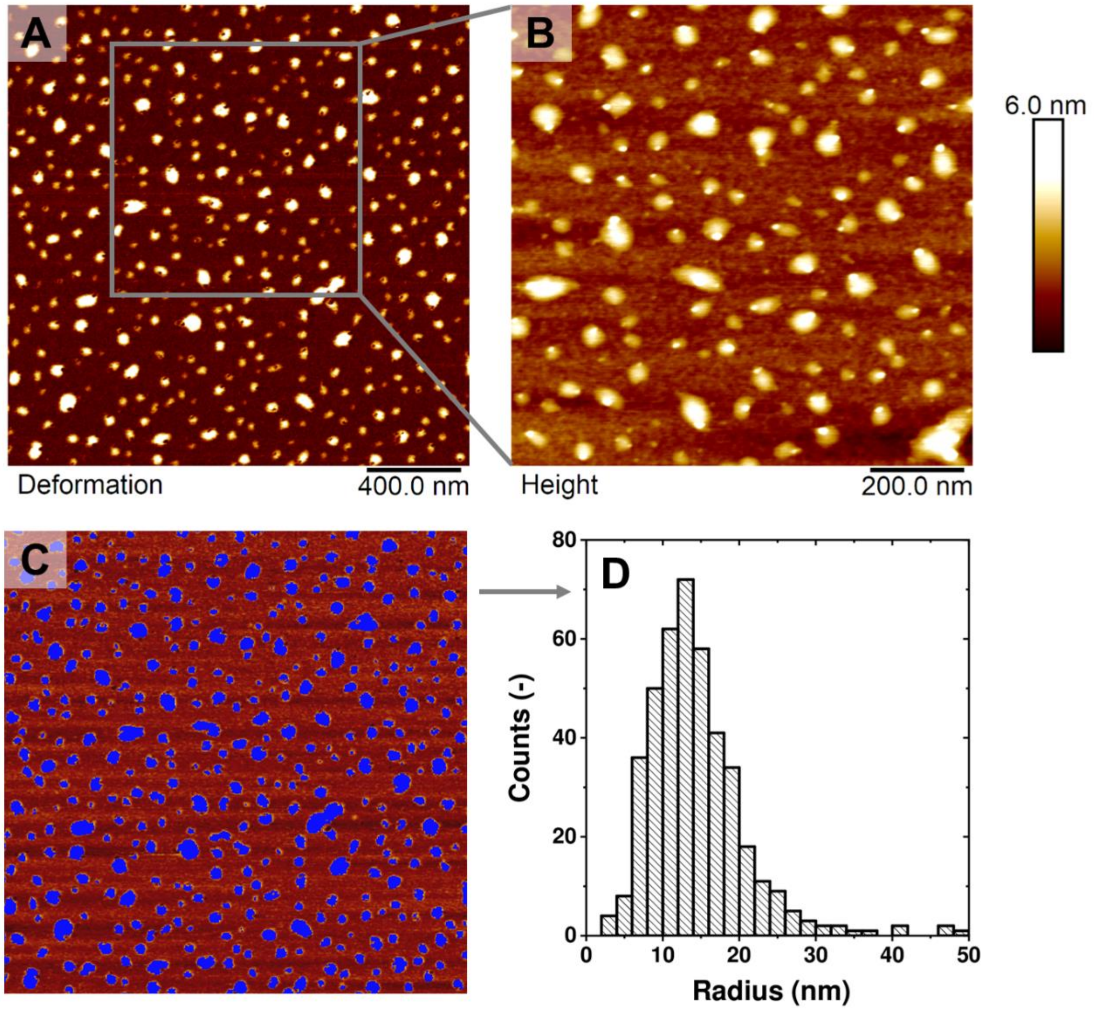

Supplementary Fig. 7. Atomic Force Microscopy (AFM) of the PPnGRAD micelles. (A) Deformation (2 µm x 2 µm) and (B) height (1 µm x 1 µm) images after spin coating on silicon wafer are shown. Image (C) represents the size analysis of the deformation image by a data threshold (cutoff). The resulting calculation is shown in (D) as a histogram. The majority of micelles had a radius of 15±7 nm (SD). In the dry state the micelles flatten on the silicon wafer resulting in larger sizes than DLS; also, some aggregates were visible, as often occurs during the drying of colloids (deposited by spin coating from aqueous dispersion c=0.2 mg mL^{-1}). Specimens were duplicated, and on each sample three independent spots were measured and showed similar results.

1.9 Stability of PPn$_{GRAD}$ micelles

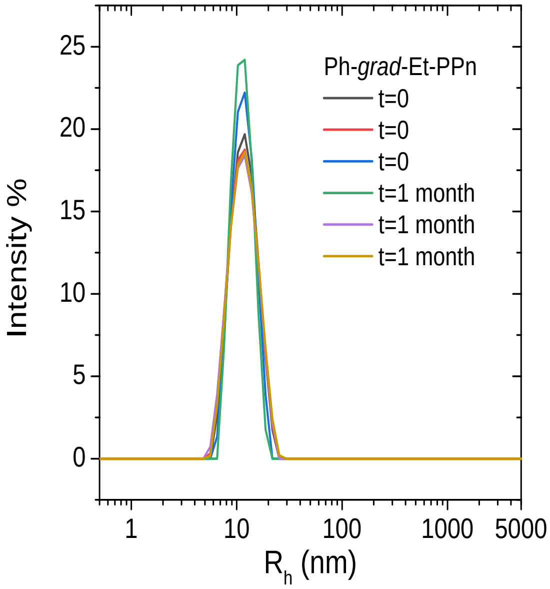

Supplementary Fig. 8. Stability of the PPn$_{GRAD}$ micelles in physiological saline solution by dynamic light scattering (DLS). The hydrodynamic radius did not change after storage for one month at 4 °C; the polydispersity index (PDI) was 0.05 at t=0 and at t=1month, confirming that no agglomeration occurred.

1.10 Viability assay with immune cells using PPn$_{GRAD}$ micelles

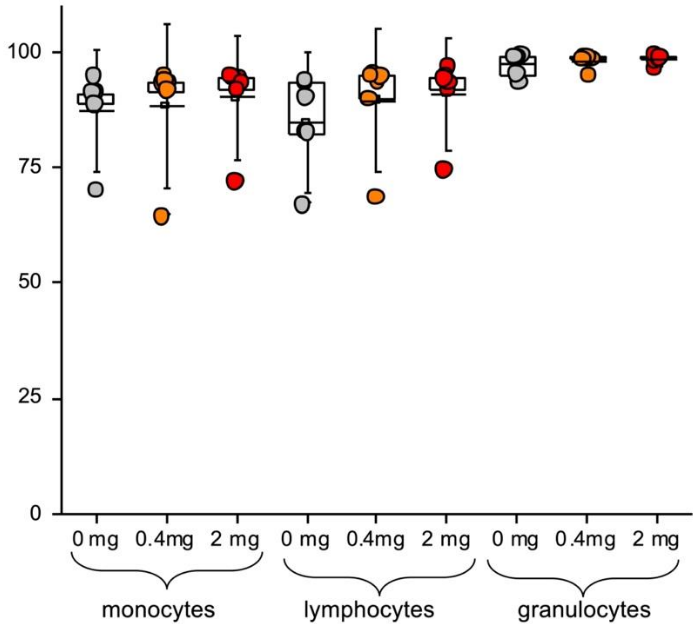

Supplementary Fig. 9. Viability of immune cells after exposure to PPn$_{GRAD}$ micelles. The micelles did not affect the survival of the cells. N=6 (data presented as mean ±SD).

Supplementary Table 3. Comparison of NMR relaxation times of different polymer batches (158 MHz, cf. Supplementary Table 1 for other polymer properties).

Batch	T_1 Et-PPn (s)	T_2 Et-PPn (s)	T_1 Ph-PPn (s)	T_2 Ph-PPn (s)
**Ph-grad-Et-PPn** batch 1 (PPn_{GRAD}) 25 °C	1.8	0.97	0.87	0.11
**Ph-grad-Et-PPn** batch 2 (PPn_{GRAD}) 25 °C	1.7	0.83	0.88	0.13
**Ph-grad-Et-PPn** batch 2 (PPn_{GRAD}) at 37 °C	2.1	0.74	0.84	0.22
**Ph-grad-Et-PPn** batch 3 (PPn_{GRAD}) 25 °C	1.8	0.98	0.87	0.14
Average (25 °C) ± standard deviation	1.8 ± 0.06	0.93 ± 0.07	0.87+0.05	0.13 ±0.01

2 ^{31}P MRI: characterization of MR properties, in vitro and in vivo imaging

2.1 Discussion of relaxation times measured by ^{31}P NMR spectroscopy

The different topologies of colloids affected their relaxation times. Particularly, the transverse relaxation T_2 of Et-PPn copolymer was different for **PPn$_{GRAD}$** and **PPn$_{SHELL}$**. Therefore, we performed a detailed analysis of T_2 results and compared different fit functions (Supplementary Fig. 10, Supplementary Table 4).

In the case **PPn$_{GRAD}$** micelles, a monoexponential decay provides a good description of the measurement results for Et-PPn copolymer at time t<1 s. Yet, some deviation, in the slow relaxation region is present (Supplementary Fig. 10 b,c, Supplementary Table 4). A biexponential fit, provides a good model through the whole measurement range (Supplementary Fig. 10 b,c, Supplementary Table 4). Conversely, in the case of **PPn$_{SHELL}$** a monoexponential decay did not provide a good fit of the experimental data (Supplementary Fig. 10 e,f, Supplementary Table 4). The results could be fit with biexponential fit at t<2 s; only the triexponential decay provided a good fit over the all measured echo times. However, shortest $T_{2,1}$ is too fast to be measured accurately with the used echo times (Supplementary Table 4).

In general, the region of the hydrophilic Et-PPn chain that is closer to the hydrophobic core shows lower mobility compared to the outer region of the chain surrounded by solvent, resulting in deviations from monoexponential decay. The increasing mobilities lead to a shorter time T_2 close to a hydrophobic core and longer T_2 in the outer region. The gradient structure results in a broader interfacial region of the micelles with mixed Ph- and Et-units compared with block copolymers. Conversely, in block-copolymers, the transition between hydrophobic and hydrophilic region is sharp. Consequently, the difference in mobility and resulting differences in transverse relaxation times are smaller in gradient micelles **PPn$_{GRAD}$** versus block copolymer micelles **PPn$_{SHELL}$**. The overall mobility of polymer chains is larger in **PPn$_{GRAD}$** leading to a longer T_2 which is advantageous for ^{31}P MRI.

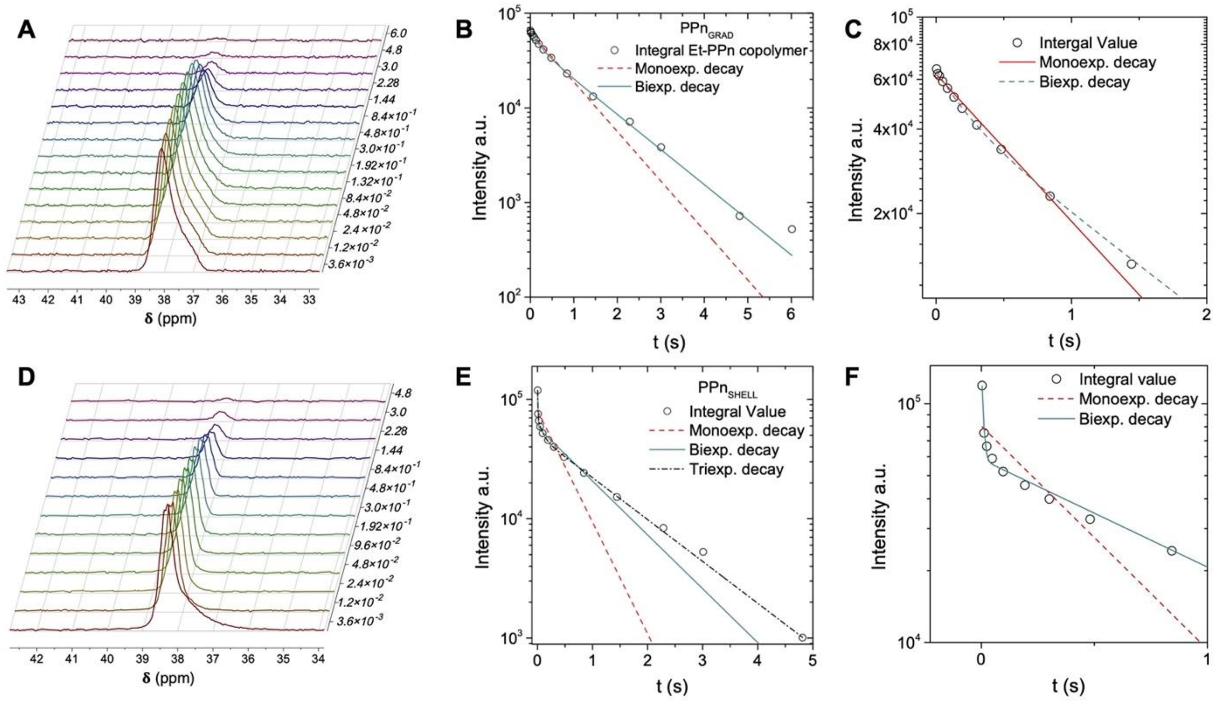

Supplementary Fig. 10. Transverse relaxation time T_2 of Et-PPn copolymer in PPn_{GRAD} and PPn_{SHELL} determined using Carr-Purcell-Meiboom-Gill Sequence (cpmg). The spectra obtained by cpmg measurement of Et-PPn copolymer in PPn_{GRAD} (A) and PPn_{SHELL} (D) are shown. Integration results and fitting of T_2 of PPn_{GRAD} (B) and PPn_{SHELL} (E) and zoom-in on the fast T_2 region (C and F). Log-lin projection was chosen to visualize the deviations of the fit. In the case of PPn_{GRAD}, monoexponential decay provided a good description of experimental data at t<1 s; splitting up the function in biexponential decay allowed for fitting of the whole array. Conversely, the PPn_{SHELL} could be fit only with a higher order exponential decay, with a triexponential fit providing the best result. These results suggest the different mobility of the Et-PPn on the surface due to different colloidal structures. 158 MHz, H_2O/D_2O 9:1 v:v.

Supplementary Table 4. Results of T_2 fitting with exponential decays of different order (A the amplitude of the fit function).

Sample	Fit-function	$T_{2,1}$ s	A_1 a.u.	$T_{2,2}$ s	A_2 a.u.	$T_{2,3}$ s	A_3 a.u.
**Ph-grad-Et-PPn**	**Monoexp.**	**0.83**	**62854**	**-**	**-**	**-**	**-**
	**Biexp.**	**0.23**	**17585**	**1.2**	**47153**	**-**	**-**
**PPn_{SHELL}**	**Monoexp.**	**0.46**	**80679**	**-**	**-**	**-**	**-**
	**Biexp.**	**0.008**	**95960**	**0.96**	**58501**	**-**	**-**
	**Triexp.**	**0.005**	**108074**	**0.08**	**22092**	**1.2**	**49440**

2.2 Determination of ^{31}P MRI signal-to-noise ratio

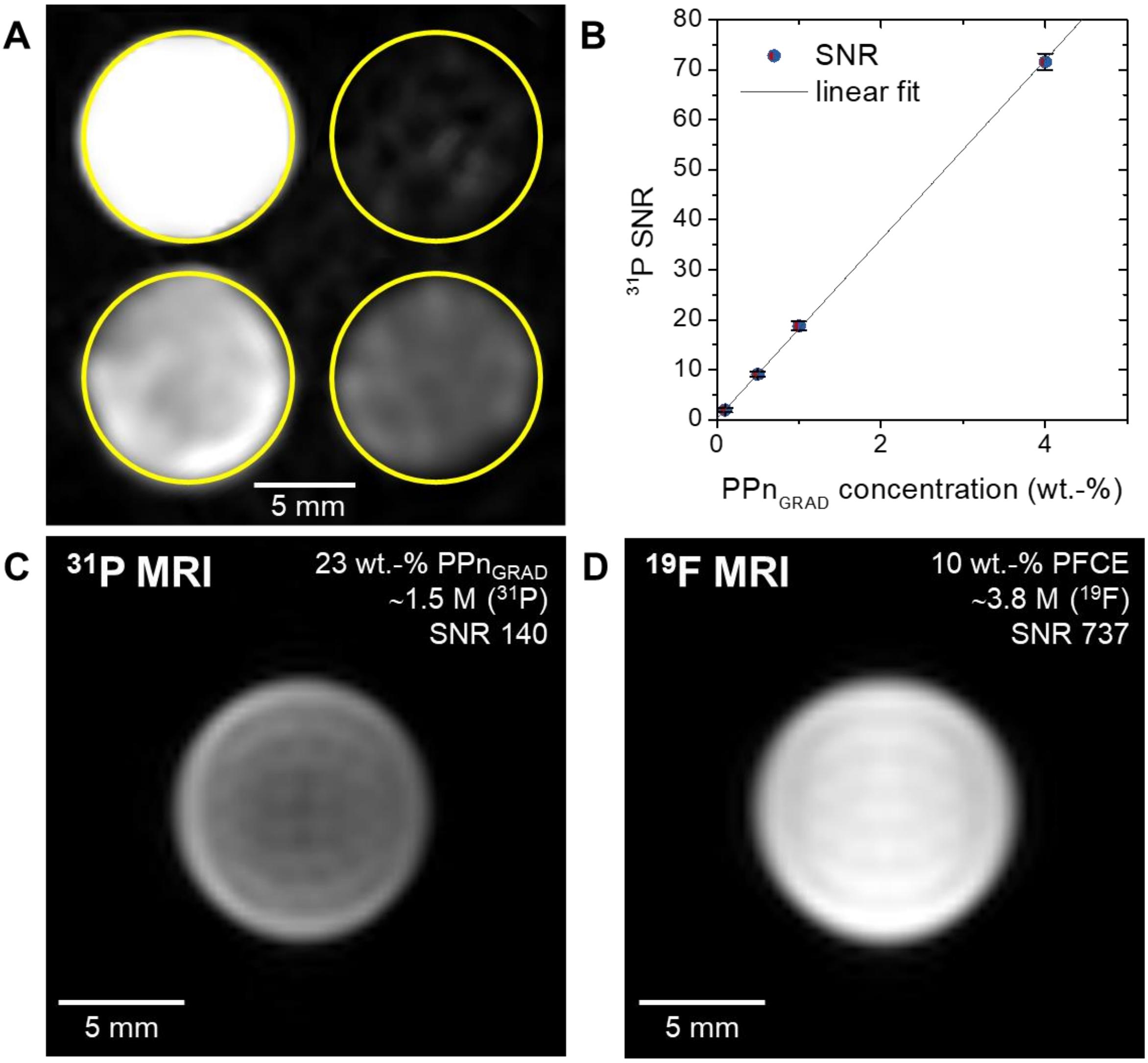

Supplementary Fig. 11. Determination of ^{31}P MRI SNR. (A) ^{31}P MRI of micelles in water containing 4, 1, 0.5 and 0.1 wt.-% PPn$_{GRAD}$ (counterclockwise from top left). (B) SNR (n=3) plotted versus concentration of PPn$_{GRAD}$ with a linear fit (R^2 = 0.99). FOV 20x20 mm^2, Matrix 64x64, Slice thickness 8 mm, TAcq 21 min 20 sec, phantom diameter 0.97 cm, 9.4 T. For SNR measurements three different spots from every image were measured (data presented as mean ±SD). (C) ^{31}P MRI of micelles in water containing 23 wt.-% PPn$_{GRAD}$, (D) ^{19}F MRI of an emulsion of perfluoro-15-crown 5 ether 10 wt.-% PFCE. FOV 20x20 mm^2, Matrix 64x64, Slice thickness 5 mm, TAcq 21 min 20 sec, phantom diameter 0.97 cm, 9.4 T.

2.3 31P MRI in vivo

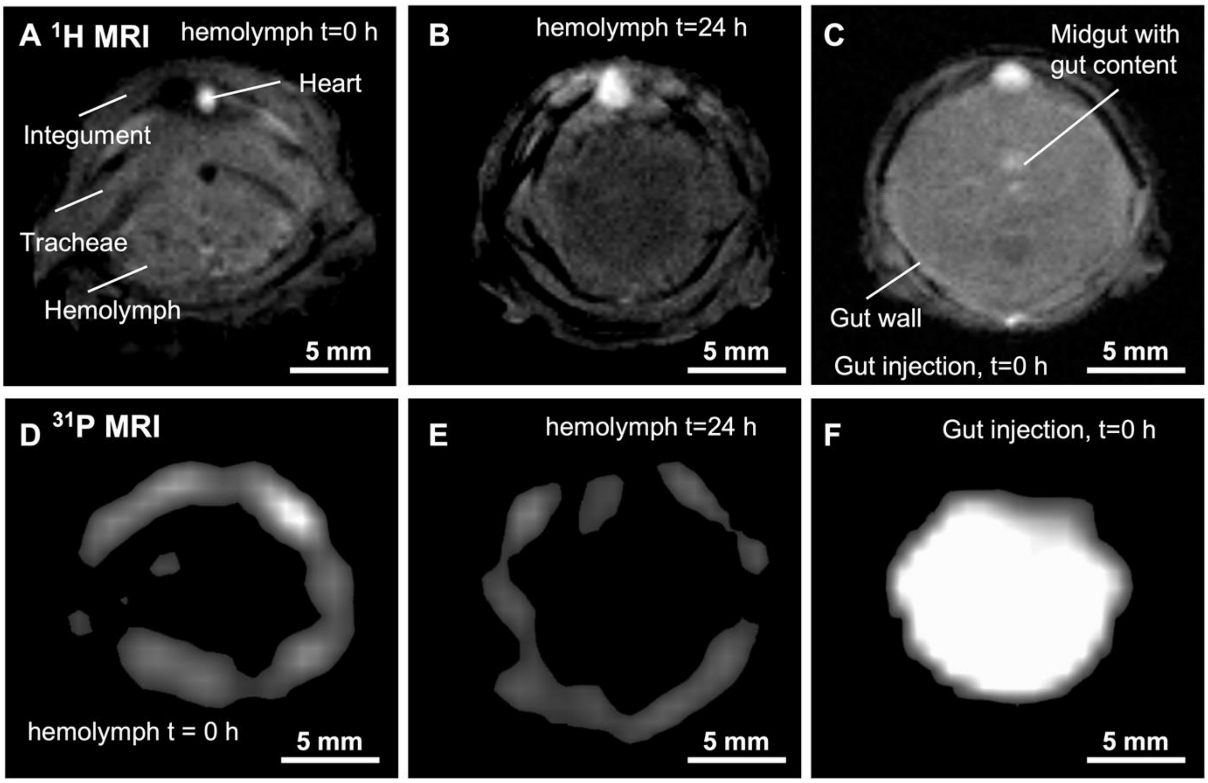

Supplementary Fig. 12. ^{1}H MRI (upper row) and corresponding ^{31}P MRI (lower row) in vivo in Manduca sexta. (A, B) ^{1}H MRI after injection of PPn$_{GRAD}$ micelles into the dorsal vessel (heart) directly (A) and 24 h (B) after injection with corresponding ^{31}P MR images (D, E) show that the micelles circulate in the hemolymph. (C and F) ^{1}H MRI and ^{31}P MRI after injection PPn$_{GRAD}$ into the gut to confirm their localization in the gut for the degradation study; after 24 h no ^{31}P signal was detected (not shown).

2.4 Degradation of PPnGRAD micelles in vivo

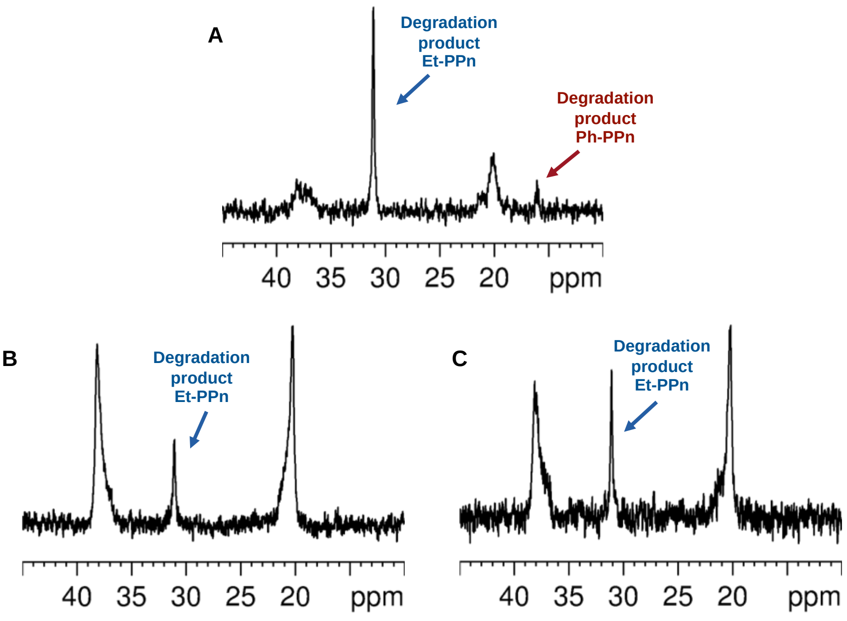

Supplementary Fig. 13. Degradation of PPnGRAD in vivo in three additional M. sexta. The ^{31}P NMR spectra of feces collected from a) M. sexta one, b) M. sexta two and c) M. sexta three 24 h after in vivo injection into the gut resuspended in D_2O are shown (158 MHz).

3 Experimental details

3.1 Materials

Solvents and chemicals were purchased from Acros Organics, Merck / Sigma-Aldrich or Fluka in at least p.a. purity. Chemicals for synthesis were additionally dried and stored over molecular sieves (4 Å), when required for synthesis (specified further in experimental procedures). Ultrapure water with a resistivity of 18.4 MΩ cm^{-1} (Milli-Q, Millipore) was used for the self-assembly experiments. 2-(Benzyloxy)ethanol was acquired from ABCR, distilled from calcium hydride and stored over molecular sieve (3 and 4 Å) and under inert gas prior to use. DBU was purchased from Sigma Aldrich, distilled from calcium hydride and stored over molecular sieves (3 and 4 Å) and under inert gas prior to use. Dulbecco's modified Eagles medium (DMEM), fetal bovine serum (FBS), penicillin-streptomycin (containing 10.000 units penicillin, 10 mg streptomycin mL^{-1}), L-Glutamine solution (sterile filtered), Trypsin-EDTA solution (sterile filtered, BioReagent), resorucin and phosphate buffered saline (PBS, pH 7.4) were purchased from Merck. Annexin V-DY-634/PI Apoptosis detection kit was purchased from Abcam.

PROTAC ARV-825 was obtained from Chemietek (USA). Deuterated solvents were purchased from Deutero GmbH (Kastellaun, Germany) and Merck and used as received.

3.2 Synthesis

3.2.1 Monomer syntheses

Synthesis of 2-Ethyl-2-oxo-1,3,2-dioxaphospholane (Et-Pn)

Et-Pn was synthesized in two steps according to the protocol from Wolf et al..[8]

Synthesis of ethyl phosphonic dichloride (EtPCl)

A mixture of diethyl-ethylphosphonate (252.9 g, 1.521 mol) and DMF (1.3 mL) were added dropwise to refluxing thionyl chloride (305 mL, 4.2 mol). Strong gas evolution of ethylene chloride and sulphur dioxide indicated the progress of the reaction. After 16 hours the gas evolution declined. To complete the reaction the bath temperature was increased to 120°C for 24 h. The thionyl chloride was separated via distillation. Two times of fractionated distillation of the raw product yielded the desired dichloride as a yellowish liquid (202.2 g, yield 100%, bp 40-42 °C/7·10^{-2} mbar).

^{1}H NMR ($CDCl_3$, ppm): δ = 2.6 (dq, $^{2}J_{HP}$ = 15.0 Hz, $^{3}J_{HH}$ = 7.5 Hz, 2H, -P-C**H**$_2$-), 1.4 (dt, $^{3}J_{HP}$ = 30.1 Hz, $^{3}J_{HH}$ = 7.5 Hz, 3H,-C**H**$_3$).

^{31}P{H} NMR ($CDCl_3$, ppm): δ = 53.7.

Synthesis of 2-Ethyl-2-oxo-1,3,2-dioxaphospholane (Et-Pn)

A flame-dried three-necked round-bottom flask, equipped with a magnetic stirring bar and two dropping funnels, was charged with 400 mL of dry THF and cooled to −21 °C. Ethylphosphonic dichloride (153.4 g, 1.04 mol) was dissolved in dry THF (400 mL) and transferred into one dropping funnel via a flame-dried stainless steel capillary. A solution of dry ethylene glycol (64.8 g, 1.04 mol) and dry pyridine (165.1 g, 2.08 mol) in THF (300 mL) was transferred into the second dropping funnel via a flame-dried stainless steel capillary. A slow dropping speed was adjusted to be approximately equal for both mixtures. After complete addition the solution was stirred for 1 h and kept overnight at −80 °C to facilitate the precipitation of the pyridinium

hydrochloride byproduct. The precipitate was removed by filtration via a flame-dried Schlenk funnel, and the solvent was removed at reduced pressure. Two times of fractionated distillation yielded the desired product as colorless oil (86.3 g, yield 61 %, bp 61 °C/ $2.1 \cdot 10^{-3}$ mbar).

^{1}H NMR ($CDCl_3$, ppm): δ = 4.6 – 4.0 (m, 4H, -CH_2-CH_2-), 1.9 (dq, $^{2}J_{HP}$ = 18.3 Hz, $^{3}J_{HH}$ = 7.8 Hz, 2H, -P-C**H**$_2$-), 1.1 (m, 3H, -C**H**$_3$).

^{31}P{H} NMR ($CDCl_3$, ppm): δ = 52.5.

Synthesis of 2-phenyl-1,3,2-dioxaphospholane 2-oxide (Ph-Pn)

Ph-Pn was synthesized according to a modified literature protocol using phenylphosphonic dichloride instead of methylphosphonic dichloride.[9] A flame-dried three-necked round-bottom flask, equipped with a magnetic stirring bar and two dropping funnels, was charged with 100 mL of dry THF and cooled to -21°C. Phenylphosphonic dichloride (50.8 g, 260 mmol) was dissolved in dry THF (250 mL) and transferred into one dropping funnel via a flame-dried stainless steel capillary. A solution of dry ethylene glycol (16.2 g, 260 mmol) and dry pyridine (41.2 g, 521 mmol) in THF (250 mL) was transferred into the second dropping funnel via a flame-dried stainless-steel capillary. A slow dropping speed was adjusted to be approximately equal for both mixtures. After complete addition, the solution was stirred for 1 hour and kept overnight at -80°C to facilitate the precipitation of the pyridinium hydrochloride byproduct. The precipitate was removed by filtration with a flame-dried Schlenk funnel and the solvent was removed at reduced pressure. Fractionated distillation yielded the desired product as colorless liquid that crystallized at room temperature (34.3 g, yield: 71 %, b.p. 113-115°C / $1 \cdot 10^{-3}$ mbar).

^{1}H NMR ($CDCl_3$, ppm, 400 MHz): δ = 7.8 (dd, $^{4}J_{HP}$ = 14.2 Hz, $^{3}J_{HH}$ = 6.9 Hz, 2H, aromatic protons ortho), 7.6-7.4 (m, 3H, aromatic protons meta, para), 4.8-4.3 (m, 4H, -O-C**H**$_2$-C**H**$_2$-O)
^{31}P{H} NMR ($CDCl_3$, ppm, 158 MHz): δ = 36.0.

3.2.2 Polymerizations

Representative procedure for the ring-opening polymerization catalyzed with DBU.

Polymerization was performed according to a modified literature protocol.[9] The particular monomers were weighed in a flame-dried Schlenk-tube, dissolved in dry benzene and dried by lyophilization. The monomer was dissolved in dry dichloromethane to a total concentration of 4 mol L^{-1}. A stock solution of initiator 2-methoxyethanol (or 2-(benzyloxy)ethanol or m-PEG_{110}) in dry dichloromethane was prepared with a concentration of 0.2 mol L^{-1} and the calculated amount was added to the monomer solution. A stock solution of DBU in dry dichloromethane was prepared with a concentration of 0.2 mol L^{-1}. The monomer solution and the catalyst solution were set to the respective reaction temperature (in general -10 °C).

The polymerization was initiated by the addition of the calculated volume of catalyst solution containing 3.0 equivalents of DBU in respect to the initiator. Polymerization was terminated by the rapid addition of an excess of formic acid dissolved in dichloromethane with a concentration of 20 mg mL^{-1}. The colorless, amorphous polymers were purified by two times precipitation in cold diethyl ether and dried in vacuo. Yields ranged from 70% to 96%.

Representative NMR data of Ph-PPn:

^{1}H NMR ($CDCl_3$, ppm, 400 MHz): δ = 7.8 – 7.6 (m, aromatic protons ortho), 7.5 (s, broad, aromatic protons para), 7.4 (s, broad, aromatic protons meta), 4.2 – 4.1 (m, backbone -CH_2-), 3.3 (d, $^{4}J_{HH}$

^{31}P{H} NMR ($CDCl_3$, ppm, 158 MHz): δ = 19.8

Representative NMR data of Ph-grad-Et-PPn:

^{1}H NMR ($CDCl_3$, ppm, 400 MHz): δ = 7.9-7.6 (m, aromatic protons ortho), 7.6-7.3 (m, aromatic protons meta, para), 4.4-3.9 (m, backbone -C**H**$_2$-), 3.30-3.24 (s, broad, initiator -C**H**$_3$), 1.9-1.5 (m, P-C**H**$_2$-), 1.3-0.9 (m, P-CH_2-<u>C**H**$_3$</u>)

^{31}P{H} NMR ($CDCl_3$, ppm, 158 MHz): δ = 35.2 (**P**-Et), 19.8 (**P**-Ph)

Real-time copolymerization kinetics

To study the incorporation behavior of the different monomers during the copolymerization, the same reaction mixture as used for polymerization was prepared and transferred into a dry NMR tube under inert gas. This mixture was used to setup all NMR parameters (i.e. shim and lock) at 263 K. The reaction was initiated by adding the calculated volume of catalyst solution (3 eq of DBU in respect to the initiator). The NMR tube was quickly pleased in the NMR spectrometer and the ^{31}P-spectra were measured at different time points to follow the polymerization. To determine the reactivity ratios, we followed the protocol of Gleede et. al. and all methods used data from 0 up to 40 % conversion to determine reactivity values. [5]

3.2.3 Preparation of Colloidal Dispersions for Imaging

Homopolymer Dispersion

The dispersion of homopolymer was prepared using miniemulsion formulation technique similarly, as described previously.[10] Ph-PPn (20 mg) was dissolved in chloroform (0.85 g) and added to an aqueous solution of sodium dodecyl sulfate (6.9 mg) in water (3.34 g). The resulting two-phase mixture was first mixed by vortexing and then sonicated in an ultrasonic bath for 1-2 min to form a pre-emulsion. Subsequently, the miniemulsion was formed using an ultrasonic sonication probe (Branson digital sonifier, 5.4 mm microtip). The miniemulsion was stirred open over night to evaporate the solvent. The excess of surfactant was removed by dialysis (regenerated cellulose membrane, molecular weight cut off 10000 g mol^{-1})

Copolymer micelles

Water-soluble copolymers were dissolved in water using a Vortexer (Fisherbrand) at a desired concentration (typically 1-15 wt.-%) yielding an opalescent dispersion.

To develop the method for encapsulation of hydrophobic payload the preparation of micelles using nanoprecipitation approach was tested. Polymer (1-4 wt.-% in relation to the final aqueous dispersion) were dissolved in 300 mg of acetone (VWR GPR recapture, 99%) and added to ultrapure water. The solution was sonicated for one minute and stirred overnight in an open vial to remove acetone.

PPn_{GRAD} Micelles loaded with PROTAC ARV-825

Micelles loaded with PROTAC ARV-825 were prepared using the second approach. Gradient copolymer (10 mg) was dissolved in acetone (280 µL) and mixed with ARV-825 (Fig 14) in acetone (0.2 mg in 20 µL acetone). Subsequently, PROTAC/Polymer solution was added to ultrapure water (490 µL), forming a clear, yellow mixture. After sonication in ultrasonic bath and evaporation overnight (controlled by weighing the vial) a yellow slightly opalescent dispersion of micelles was obtained.

As a control, the procedure was repeated without adding the polymer; ARV-825 (0.2 mg in 20 mL acetone) was added to ultrapure water. The precipitation of ARV-825 was observed immediately after the addition of the acetone solution into water.

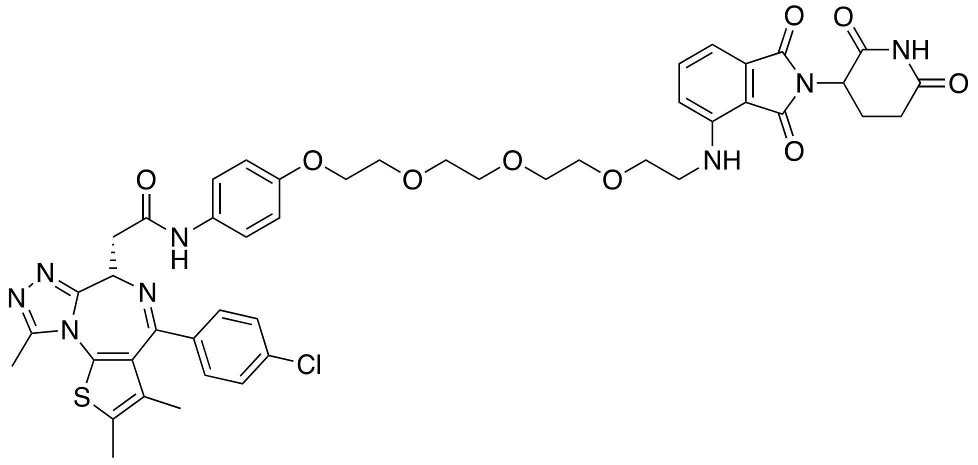

Supplementary Fig. 14 Chemical structure of PROTAC ARV-825.

3.3 Magnetic Resonance Imaging

MRI data were recorded at vertical Bruker AVANCE^{III} and AVANCE NEO 9.4 T wide bore NMR spectrometers driven by ParaVision 5.1 and 360 v3.2, respectively, and operating at frequencies of 400.2 MHz for ^{1}H and 162.0 MHz for ^{31}P measurements. Experiments were carried out using a Bruker microimaging unit (Micro 2.5) equipped with actively shielded gradient sets (capable of 1.5 T/m maximum gradient strength and 150 µs rise time at 100% gradient switching) and a dual tunable ^{1}H/^{31}P 25-mm birdcage resonator.

^{1}H and ^{31}P MRI of aqueous dispersion phantoms. Aqueous dispersions were used at a concentration of polymer of 38 mg mL^{-1}. Multi-chemical selective imaging (mCSSI) was carried out as described previously[11] using selective exciting frequencies with a bandwidth of 913 Hz (gauss 3 ms): For PPn_{GRAD} 163.0138883 and 162.0110640 MHz, PPn_{CORE} 162.0107971 MHz, PPn_{SHELL} 162.0140139 MHz, TE 6.32 ms, TR 2.5 sec, RARE factor 32, matrix 32×32, ST 8 mm, effective spectral bandwidth 15000 Hz, NA 410, TAcq 17 min 5 s.

In vivo ^{31}P MRI. Larvae were anaesthetized with 1.5% isoflurane using a home-build nose cone and fixed within the animal handling system. This was easily tolerated by the moths, which awaked 1-2 minutes after removal of the inhalation anesthesia. The larvae were and injected with PPn_{GRAD} micelles (15 mg) in physiological saline (100 µL) into the dorsal vessel or the anterior gut after anesthetizing them. Due to the vertical orientation of the MRI system, all phantoms and animals were scanned in an upright position.

Anatomical reference images were acquired by standard FLASH or RARE (FLASH: FOV 20×20 mm^2, matrix 128×128, TE 1.62 ms, TR 60 ms, ST 2 mm, NA 1, TAcq 8 sec; RARE: FOV 20×20 mm^2, matrix 256×256, TE 4.39 ms, TR 4 sec, ST 0.75 mm, RARE factor 4, NA 1, TAcq 4 min 16 sec). Subsequently, different the injected PPn_{GRAD} micelles were detected by mCSSI. The slice used for ^{31}P MRI (6-10 mm) was placed in axial orientation covering a large part of the physalis or larvae. mCSSI: 163.0138883 and 162.0110640 MHz, effective TE 46 ms, TR 500 ms, RARE factor 16, FOV 20×20 mm2, matrix 32×32, effective spectral bandwidth 15000 Hz, NA 2048, TAcq 17 min 5 s.

3.4 Cell Experiments

Viability assay in immune cells. To obtain circulating immune cells, heparinized blood was withdrawn by venous puncture of the inferior vena cava of male 12 – 14 weeks old C57bl/6j mice (file reference O19/99). Blood was collected via a 23G cannula in heparin-aerated collection tubes. Erythrocytes were lysed by adding the 4-fold amount of ammonium chloride buffer (pH 7.4). After 10 min of incubation at room temperature the samples were centrifuged at 350 g for 10 min at 20 °C.

To determine cell toxicity of Ph-grad-Et-PPn micelles, 1x10^6 murine immune cells were incubated with either 10 or 50µl/ml in DMEM and incubated for 1 h at 37 °C. Afterwards, cells were washed with 200 µL MACS buffer and stained with CD45 BD Bioscience, clone HK1.4), CD11b (BioLegend, clone M1/70) and Ly6G (BD Bioscience, clone 1A8) all 1:100 in 100 µl MACS buffer to discriminate monocytes (CD45$^+$, CD11b$^+$, Ly6G$^-$), lymphocytes (CD45$^+$, CD11b$^-$, Ly6G$^-$) and granulocytes (CD45$^+$, CD11b$^+$, Ly6G$^+$). Flow cytometry was performed with a FACSCanto II (BD Biosciences). Cells were gated with appropriate FSC–SSC settings and thresholds for excluding debris. To omit dead cells, samples were stained with 1 µg ml^{-1} DAPI (Merck). For analysis, cells were gated with FlowJo 7.1, and the number of DAPI negative cells was determined.

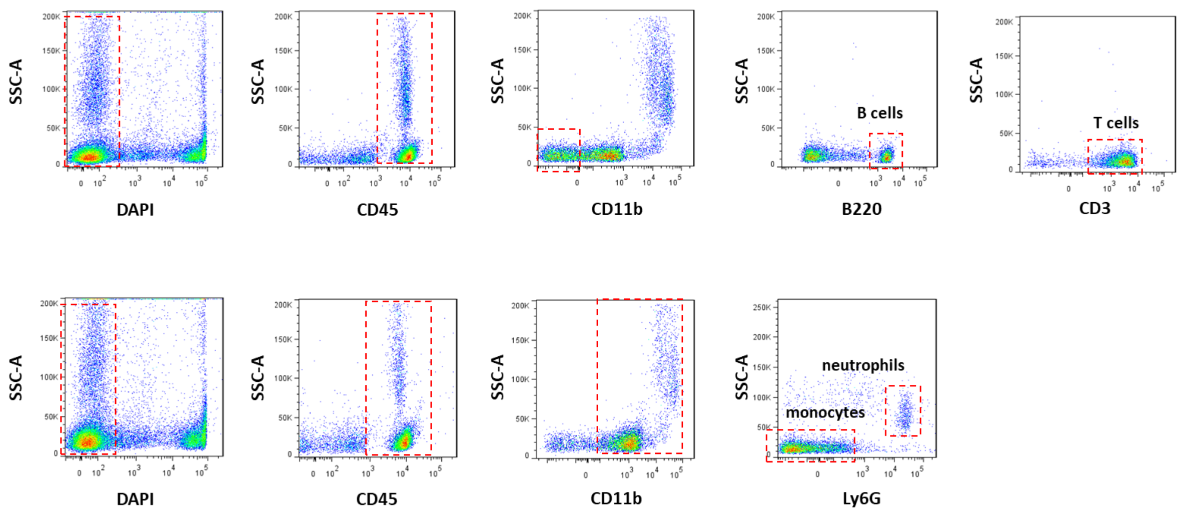

Supplementary Fig. 15. Murine immune cell gating strategy

Viability assay of cancer cells treated with PROTAC-loaded Ph-grad-Et-PPn micelles. HeLa cells were seeded at 7.5×10^3 cells per well in 100 µL medium in 96-well plates and incubated overnight at 37 °C in humidified 5% CO_2-containing atmosphere. Sample solutions of 1, 5 and 10 µM ARV-825 were prepared using PROTAC-loaded micelles or ARV-825 stock solution in DMSO. The medium was aspirated from the wells and 100 µL of the sample medium was added per well in triplicates. In the reference wells 100 µL medium was added. The samples were incubated for 72 h before the cells were washed with PBS. A resazurin assay

was used to analyze the viability of the cells, 100 µL of resazurin solution was added per well and incubated for 1 h. The fluorescence intensity was measured by a plate reader (EnSpire) at excitation and emission wavelength of 560/590 nm.

Flow cytometry for apoptosis analysis.

HeLa cells (up to p.20) were cultured in DMEM medium with 10% FBS, L-glutamine and Pen/Strep. The cells were seeded at 15 × 10^3 cells per well in 300 µL medium in 48 well plates and incubated overnight at overnight at 37 °C in humidified 5% CO_2-containing atmosphere. From a stock solution of micelles $conc_{polymer}$ 22 mg mL^{-1} and $conc_{ARV}$ 487 µM sample solution of 1 µM ARV-825 were prepared. ARV-825 loaded micelles, unloaded micelles and free ARV-825 (diluted from DMSO stock) were incubated at 1 µM of ARV-825 on HeLa cells for 72 h. Then HeLa cells were harvested with trypsin and the samples were prepared for flow cytometry by using an Annexin V-DY-434/PI Apoptosis detection kit. The ARV-825 activity on HeLa cells was analyzed using BD Bioscience FACS Aria II with excitation and emission filter of 375-450/30, 488-530/30 and 630-660/30 nm (software- BDFACS Diva software).

Gating strategy: The cell population was gated using reference HeLa cells in the FSC/SSC plot. The background of Annexin V (excitation/emission 633-660/20 nm) and PI (excitation/emission 488- 530/30 nm) were determined using reference HeLa cells and the corresponding gates were used throughout the experiment to determine positive cell population for each staining.

References

1. Jaacks V. A novel method of determination of reactivity ratios in binary and ternary copolymerizations. Die Makromolekulare Chemie: Macromolecular Chemistry and Physics 1972, **161**(1)**:** 161-172.

2. Beckingham BS, Sanoja GE, Lynd NA. Simple and accurate determination of reactivity ratios using a nonterminal model of chain copolymerization. Macromolecules 2015, **48**(19)**:** 6922-6930.

3. Blankenburg J, Kersten E, Maciol K, Wagner M, Zarbakhsh S, Frey H. The poly (propylene oxide-co-ethylene oxide) gradient is controlled by the polymerization

method: determination of reactivity ratios by direct comparison of different copolymerization models. Polymer Chemistry 2019, **10**(22)**:** 2863-2871.

4. Meyer VE, Lowry GG. Integral and differential binary copolymerization equations. Journal of Polymer Science Part A: General Papers 1965, **3**(8)**:** 2843-2851.

5. Gleede T, Markwart JC, Huber N, Rieger E, Wurm FR. Competitive Copolymerization: Access to Aziridine Copolymers with Adjustable Gradient Strengths. Macromolecules 2019, **52**(24)**:** 9703-9714.

6. Sedlacek O, Lava K, Verbraeken B, Kasmi S, De Geest BG, Hoogenboom R. Unexpected Reactivity Switch in the Statistical Copolymerization of 2-Oxazolines and 2-Oxazines Enabling the One-Step Synthesis of Amphiphilic Gradient Copolymers. Journal of the American Chemical Society 2019, **141**(24)**:** 9617-9622.

7. Kabanov AV, Batrakova EV, Alakhov VY. Pluronic® block copolymers as novel polymer therapeutics for drug and gene delivery. Journal of Controlled Release 2002, **82**(2)**:** 189-212.

8. Wolf T, Steinbach T, Wurm FR. A Library of Well-Defined and Water-Soluble Poly(alkyl phosphonate)s with Adjustable Hydrolysis. Macromolecules 2015, **48**(12)**:** 3853-3863.

9. Steinbach T, Ritz S, Wurm FR. Water-Soluble Poly(phosphonate)s via Living Ring-Opening Polymerization. ACS Macro Letters 2014, **3**(3)**:** 244-248.

10. Alexandrino EM, Ritz S, Marsico F, Baier G, Mailänder V, Landfester K, et al. Paclitaxel-loaded polyphosphate nanoparticles: a potential strategy for bone cancer treatment. Journal of Materials Chemistry B 2014, **2**(10)**:** 1298-1306.

11. Flögel U, Temme S, Jacoby C, Oerther T, Keul P, Flocke V, et al. Multi-targeted 1H/19F MRI unmasks specific danger patterns for emerging cardiovascular disorders. Nature Communications 2021, **12**(1)**:** 5847.
