# Supplementary material for: Biodegradable polyphosphoester micelles act as both background-free 31P magnetic resonance imaging agents and drug nanocarriers
Source: Nat Commun. 2023 Jul 19;14:4351. doi: 10.1038/s41467-023-40089-0 (PMC10356825; doi:10.1038/s41467-023-40089-0)
Supplement: Supplementary file 2 — Reporting Summary [file 41467_2023_40089_MOESM2_ESM.pdf]

nature portfolio

Corresponding author(s): Koshkina, Wurm, Flögel
Last updated by author(s): July, 05, 2023

Reporting Summary

Nature Portfolio wishes to improve the reproducibility of the work that we publish. This form provides structure for consistency and transparency in reporting. For further information on Nature Portfolio policies, see our Editorial Policies and the Editorial Policy Checklist.

Statistics

For all statistical analyses, confirm that the following items are present in the figure legend, table legend, main text, or Methods section.

n/a	Confirmed	
☐	☒	The exact sample size (*n*) for each experimental group/condition, given as a discrete number and unit of measurement
☐	☒	A statement on whether measurements were taken from distinct samples or whether the same sample was measured repeatedly
☐	☒	The statistical test(s) used AND whether they are one- or two-sided<br>*Only common tests should be described solely by name; describe more complex techniques in the Methods section.*
☒	☐	A description of all covariates tested
☐	☒	A description of any assumptions or corrections, such as tests of normality and adjustment for multiple comparisons
☐	☒	A full description of the statistical parameters including central tendency (e.g. means) or other basic estimates (e.g. regression coefficient) AND variation (e.g. standard deviation) or associated estimates of uncertainty (e.g. confidence intervals)
X		For null hypothesis testing, the test statistic (e.g. *F*, *t*, *r*) with confidence intervals, effect sizes, degrees of freedom and *P* value noted<br>Give P values as exact values whenever suitable.
☐	☒	For Bayesian analysis, information on the choice of priors and Markov chain Monte Carlo settings
☒	☐	For hierarchical and complex designs, identification of the appropriate level for tests and full reporting of outcomes
X		Estimates of effect sizes (e.g. Cohen's *d*, Pearson's *r*), indicating how they were calculated

Our web collection on statistics for biologists contains articles on many of the points above.

Software and code

Policy information about availability of computer code

Data collection	TopSpin 8, ParaVision 5.1, Trios DSC Software 5, BDFACS Diva software, FlowJo 7.1
Data analysis	MestreNova 10.0.2, Origin 2019, MatLab 8, ParaVision 5.1, LabVIEW 6, Amira 4.0, FlowJo, Microtrac Flex Software, Fiji ImageJ v2.1.0, Cell Counter (2010/12/07), Raptor X (2021/06), BDFACS Diva software

For manuscripts utilizing custom algorithms or software that are central to the research but not yet described in published literature, software must be made available to editors and reviewers. We strongly encourage code deposition in a community repository (e.g. GitHub). See the Nature Portfolio guidelines for submitting code & software for further information.

Data

Policy information about availability of data

All manuscripts must include a data availability statement. This statement should provide the following information, where applicable:

- Accession codes, unique identifiers, or web links for publicly available datasets
- A description of any restrictions on data availability
- For clinical datasets or third party data, please ensure that the statement adheres to our policy

All data necessary to understand and assess the conclusions of the manuscript are present in the paper or the supplementary materials; all raw data can be made available on request by the authors.

Human research participants

Policy information about studies involving human research participants and Sex and Gender in Research.

Reporting on sex and gender	n/A
Population characteristics	n/A
Recruitment	n/A
Ethics oversight	n/A

Note that full information on the approval of the study protocol must also be provided in the manuscript.

Field-specific reporting

Please select the one below that is the best fit for your research. If you are not sure, read the appropriate sections before making your selection.

☒ Life sciences ☐ Behavioural & social sciences ☐ Ecological, evolutionary & environmental sciences

For a reference copy of the document with all sections, see nature.com/documents/nr-reporting-summary-flat.pdf

Life sciences study design

All studies must disclose on these points even when the disclosure is negative.

Sample size	No statistical methods were used to predetermine sample size. The sample size was sleected based on common research standards in the polyme rscience / nanotechnology field (e.g. n= 3 or n= 6 for toxicity testing)
Data exclusions	No data were excluded.
Replication	Instead of replication with the same cell type, the toxicity test were carried out with three different cell types providing similar results
Randomization	Larvae were chosen randomly from the colony (= allocation was random within the required developmental age margin). Furthermore, no different treatment groups were involved. The Manduca Sexta Model is well-known in literature, furthermore, no different treatment groups were involved. In the present study, we aimed to characterize detection of 31P-containing polymers by non-invasive MRI techniques. Randomization is not applicable to the other experiments
Blinding	Blinding was not feasible because the studied phenotypes allowed unambiguous identification of the treatment in most cases. The Manduca Sexta Model is well-known in literature, furthermore, no different treatment groups were involved. In the present study, we aimed to characterize detection of 31P-containing polymers by non-invasive MRI techniques. Thus, we felt that no randomization or blinding of animals was required. Blinding is not necessary / applicable for the other experiment

Reporting for specific materials, systems and methods

We require information from authors about some types of materials, experimental systems and methods used in many studies. Here, indicate whether each material, system or method listed is relevant to your study. If you are not sure if a list item applies to your research, read the appropriate section before selecting a response.

Materials & experimental systems

n/a	Involved in the study	
x	☐	Antibodies
☐	☒	Eukaryotic cell lines
☒	☐	Palaeontology and archaeology
☐	☒	Animals and other organisms
☒	☐	Clinical data
☒	☐	Dual use research of concern

Methods

n/a	Involved in the study	
☒	☐	ChIP-seq
☐	☒	Flow cytometry
☒	☐	MRI-based neuroimaging

Eukaryotic cell lines

Policy information about cell lines and Sex and Gender in Research

Cell line source(s)	HeLa from ECACC (European collection of authenticated Cell culture) purchased from Sigma.
Authentication	We did not do any further authentication or mycoplasma contamination tests on the HeLa cells.
Mycoplasma contamination	not used
Commonly misidentified lines (See ICLAC register)	not used

Animals and other research organisms

Policy information about studies involving animals; ARRIVE guidelines recommended for reporting animal research, and Sex and Gender in Research

Laboratory animals	The Manduca sexta larvae were obtained from our in-house colony, which has now existed for over 25 years at the University of Giessen. Only animals of the same developmental stage (L5 larvae, day 5–6 if not indicated otherwise) were included in this study. For murine experiments, male 10 to 12-week-old C57Bl/6 mice ranging from 20 to 30 g body weight (BW) were used. Animals used in this study were obtained from Janvier, housed at the central animal facility of the Heinrich-Heine-Universität Düsseldorf (ZETT, Düsseldorf, Germany) on a 12 hrs light/dark cycle, fed with a standard chow diet and received tap water ad libitum.
Wild animals	This study does not involve wild animals.
Reporting on sex	Male animals were used, gender is not relevant for this ecxperiments.
Field-collected samples	The study did not involve samples collected from the field.
Ethics oversight	No ethical approval or guidance was required to use Manduca sexta as a model system. C57bl/6j mice (file reference O19/99) only blood was used, not requiring ethical approval.

Note that full information on the approval of the study protocol must also be provided in the manuscript.

Flow Cytometry

Plots

Confirm that:

☒ The axis labels state the marker and fluorochrome used (e.g. CD4-FITC).

☒ The axis scales are clearly visible. Include numbers along axes only for bottom left plot of group (a 'group' is an analysis of identical markers).

☒ All plots are contour plots with outliers or pseudocolor plots.

☒ A numerical value for number of cells or percentage (with statistics) is provided.

Methodology

Sample preparation	HeLa cells were cultured and harvested as described in the methods, HeLa cells were stained with Annexin V and PI for the experiment.
Instrument	BDFACS Aria II
Software	BDFACS Diva software
Cell population abundance	10 000 events were measured of the gated population per sample.
Gating strategy	The cell population was gated using reference HeLa cells in the FSC/SSC plot. The background of Annexin V (excitation/emission 633-660/20 nm) and PI (excitation/emission 488- 530/30 nm) were determined using reference HeLa cells and the corresponding gates were used throughout the experiment to determine positive cell population for each staining.

☒ Tick this box to confirm that a figure exemplifying the gating strategy is provided in the Supplementary Information.
